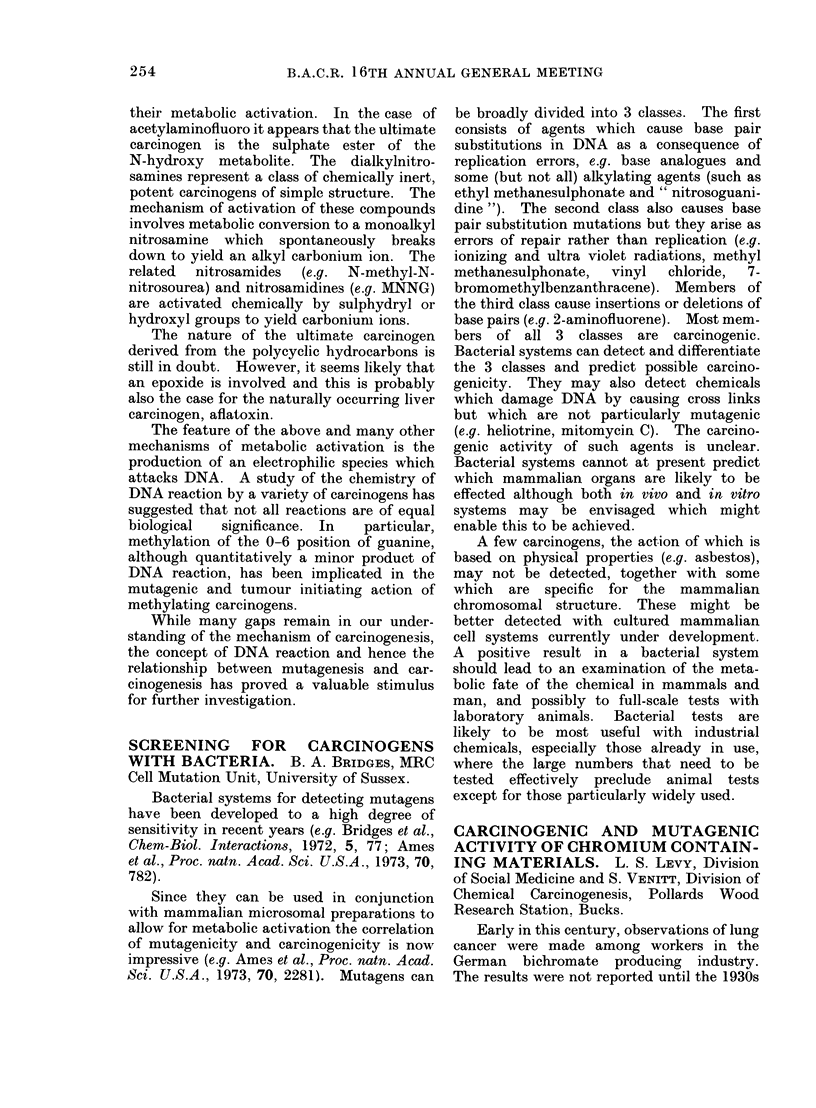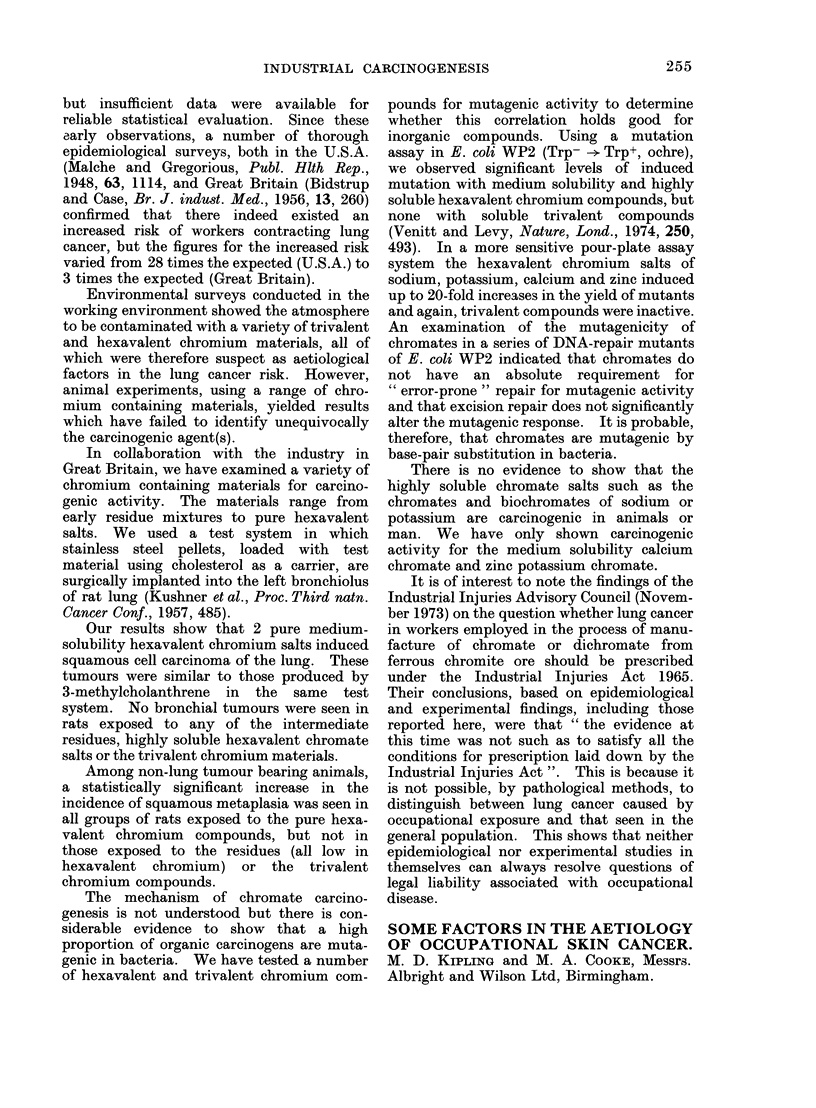# Proceedings: Carcinogenic and mutagenic activity of chromium containing materials.

**DOI:** 10.1038/bjc.1975.201

**Published:** 1975-08

**Authors:** L. S. Levy, S. Venitt


### CARCINOGENIC AND MUTAGENIC ACTIVITY OF CHROMIUM CONTAINING MATERIALS.

L. S. Levy, Division of Social Medicine and S. Venitt, Division of Chemical Carcinogenesis, Pollards Wood Research Station, Bucks.

Early in this century, observations of lung cancer were made among workers in the German bichromate producing industry. The results were not reported until the 1930s but insufficient data were available for reliable statistical evaluation. Since these early observations, a number of thorough epidemiological surveys, both in the U.S.A. (Malche and Gregorious, *Publ. Hlth Rep.*, 1948, **63**, 1114, and Great Britain (Bidstrup and Case, *Br. J. indust. Med.*, 1956, **13**, 260) confirmed that there indeed existed an increased risk of workers contracting lung cancer, but the figures for the increased risk varied from 28 times the expected (U.S.A.) to 3 times the expected (Great Britain).

Environmental surveys conducted in the working environment showed the atmosphere to be contaminated with a variety of trivalent and hexavalent chromium materials, all of which were therefore suspect as aetiological factors in the lung cancer risk. However, animal experiments, using a range of chromium containing materials, yielded results which have failed to identify unequivocally the carcinogenic agent(s).

In collaboration with the industry in Great Britain, we have examined a variety of chromium containing materials for carcinogenic activity. The materials range from early residue mixtures to pure hexavalent salts. We used a test system in which stainless steel pellets, loaded with test material using cholesterol as a carrier, are surgically implanted into the left bronchiolus of rat lung (Kushner *et al.*, *Proc. Third natn. Cancer Conf.*, 1957, 485).

Our results show that 2 pure medium-solubility hexavalent chromium salts induced squamous cell carcinoma of the lung. These tumours were similar to those produced by 3-methylcholanthrene in the same test system. No bronchial tumours were seen in rats exposed to any of the intermediate residues, highly soluble hexavalent chromate salts or the trivalent chromium materials.

Among non-lung tumour bearing animals, a statistically significant increase in the incidence of squamous metaplasia was seen in all groups of rats exposed to the pure hexavalent chromium compounds, but not in those exposed to the residues (all low in hexavalent chromium) or the trivalent chromium compounds.

The mechanism of chromate carcinogenesis is not understood but there is considerable evidence to show that a high proportion of organic carcinogens are mutagenic in bacteria. We have tested a number of hexavalent and trivalent chromium compounds for mutagenic activity to determine whether this correlation holds good for inorganic compounds. Using a mutation assay in *E. coli* WP2 ($Trp^- \rightarrow Trp^+$, ochre), we observed significant levels of induced mutation with medium solubility and highly soluble hexavalent chromium compounds, but none with soluble trivalent compounds (Venitt and Levy, *Nature, Lond.*, 1974, **250**, 493). In a more sensitive pour-plate assay system the hexavalent chromium salts of sodium, potassium, calcium and zinc induced up to 20-fold increases in the yield of mutants and again, trivalent compounds were inactive. An examination of the mutagenicity of chromates in a series of DNA-repair mutants of *E. coli* WP2 indicated that chromates do not have an absolute requirement for " error-prone " repair for mutagenic activity and that excision repair does not significantly alter the mutagenic response. It is probable, therefore, that chromates are mutagenic by base-pair substitution in bacteria.

There is no evidence to show that the highly soluble chromate salts such as the chromates and biochromates of sodium or potassium are carcinogenic in animals or man. We have only shown carcinogenic activity for the medium solubility calcium chromate and zinc potassium chromate.

It is of interest to note the findings of the Industrial Injuries Advisory Council (November 1973) on the question whether lung cancer in workers employed in the process of manufacture of chromate or dichromate from ferrous chromite ore should be prescribed under the Industrial Injuries Act 1965. Their conclusions, based on epidemiological and experimental findings, including those reported here, were that " the evidence at this time was not such as to satisfy all the conditions for prescription laid down by the Industrial Injuries Act ". This is because it is not possible, by pathological methods, to distinguish between lung cancer caused by occupational exposure and that seen in the general population. This shows that neither epidemiological nor experimental studies in themselves can always resolve questions of legal liability associated with occupational disease.